# Carbon Stable-Isotope and Physicochemical Data as a Possible Tool to Differentiate between Honey-Production Environments in Uruguay

**DOI:** 10.3390/foods7060086

**Published:** 2018-06-06

**Authors:** Verónica Berriel

**Affiliations:** Centre for Applications of Nuclear Technology in Sustainable Agriculture, Soil and Agronomy College, University of the Republic, Av. Garzón 809, CP 12.900 Montevideo, Uruguay; vberriel@fagro.edu.uy; Tel.: +598-2356-4488

**Keywords:** honey, physicochemical parameters, C isotope composition, discriminant analysis

## Abstract

The allocation of honey origin is an increasingly important issue worldwide as it is closely related to product quality and consumer preference. In South America, honeys produced in grasslands and eucalyptus or native forests are preferred at the regional level, so their differentiation is essential to assure consumers of their authenticity according to their productive system. The objective of this study was to differentiate honeys produced in three environments: one, a monoculture system based on the eucalyptus forest, and two others based in natural environments of grasslands and native forests. To do this, honey’s physicochemical and isotopic variables (pH, free acidity, lactic acid content, moisture, total sugar content, and honey and extracted protein ^13^C isotopic composition) were analysed. Discriminant analysis applied to the data revealed that, based on the selected variables, it was impossible to differentiate the three groups of honeys due to the superposition of those produced in grasslands and native forests. For this reason, a group of honeys derived from native and polyfloral environments (grasslands and native forests) was formed and subjected to discriminant analysis (DA), together with the group of honeys derived from a commercial plantation of eucalyptus forest. The model obtained in this case achieved 100% correct allocation both at the training stage and the cross-validation stage.

## 1. Introduction

Nowadays, there is a large group of consumers who prefer to buy food based on its attributes, including its production system. These consumers demand more than the market guarantees regarding the origin of the food and certification of the type of system in which it was produced (for example, in the form of green [1], sustainable production [2,3,4], etc.). For this reason, it is increasingly necessary to develop tools to authenticate agri-food production systems. An interesting case study in Uruguay involves the production of honey. In this country, beekeeping is based on intensive farming systems, such as monocultures (for example, forest production) or on production in environments where multiple native or naturalised species coexist (for example, in native forests or grasslands) [5]. Differentiating between the honeys produced in different systems could be important for sellers and consumers.

The vast majority of investigations related to the assignment of origin of honey have been restricted to botanical or geographical origin [6], and to a much lesser extent to the organic provenance (free of heavy metals, radioactive isotopes, organic pollutants, pesticides, pathogenic bacteria, and pollen of genetically modified organisms) [6]. Little information is available about studies on the origin of honey according to its production system. The differentiation of origin can be done through chemometric analysis because the chemical composition of honey depends not only on the type of flower visited by the bee, but also on the climatic conditions and soil type of the location at which the plants grew [7,8,9].

In this regard, many studies have demonstrated that it is possible to differentiate honeys according to their floral or geographical origin [4]. The chemical variables used in research have been varied from those that define the quality of the product, such as pH, acidity or moisture, to more specific ones, such as mineral content [4], or spectral information derived from the use of visible (vis) and near infrared (NIR) spectroscopy [10] or the stable elements’ isotopic composition [11]. Combinations of these variables have been used in conjunction with multivariate statistical methods.

For example, a 100% correct classification of honey samples according to their botanical background (*Eucalyptus*, *Citrus*, *Lythrum*, *Umbelliferae* or honeydew) was achieved in a study carried out in Morocco [12] that applied principal component analyses (PCA) and discriminant analyses (DA) to a set of physicochemical data (colour, moisture content, pH, free and lactonic acidity, hydroxymethylfurfural (HMF), diastolic activity, electrical conductivity, mineral concentration and sugar content).

A similar success rate was obtained in Spain by Nozal-Nalda et al. [13], who employed PCA and DA (linear and quadratic) to identify honeys produced in different floral areas. The predominant species in each of these areas included French lavender, spike lavender, rosemary, ling, heather, honeydew and thyme, while the analysed physicochemical variables comprised ash and insoluble matter content (%), mineral concentration, reducing sugar content, diastase activity, free acidity and HMF content. The authors found that the variables with the greatest discriminant power according to DA were free acidity, reducing sugar content, Ca and Na.

In Italy, Bontempo et al. [11] achieved good separation of honey according to floral origin (*Acacia*, chestnut, *Citrus*, *Eucalyptus*, honeydew and *Rhododendron*). These authors applied PCA, using variables related to the mineral composition of the honeys and the isotopic signatures (C, N and H) of their proteins.

In Uruguay, Corbella and Cozzolino [5] performed DA on several physicochemical variables (moisture content, pH, electrical conductivity, HMF content and colour). They reported correct classification of more than 80% of honey samples, differentiating those originating from planted pastures (lotus, clover and lucerne), citrus plantations and *Baccharis*. These same authors also used chemometrics to achieve the perfect classification of pasture and eucalyptus honeys from Uruguay based on the spectral information derived from the use of vis and NIR spectroscopy through partial least-squares–discriminant analysis (PLS–DA) [10].

Uruguay is an important global exporter of honey, most of which is produced in exotic eucalyptus forest (commercial plantations), but also includes highly natural and polyfloral honey produced in grasslands and native forests. To improve the competitiveness of Uruguayan honey, the aim of this study was to establish the quality of honeys produced in eucalyptus forests, grasslands and native forests, and to assess the possibility of discriminating between productive systems. This differentiation by origin was based on the combination of simple physicochemical and isotopic carbon composition variables modelled by discriminant analyses.

## 2. Materials and Methods

### 2.1. Collection of Samples

Honey samples were collected from three environments (eucalyptus forest, grassland and native forest) from 15 apiaries located in different areas of Uruguay (30° and 35° S, 53° and 58° W). Apiary selection was based on an attempt to represent the geographical variability of the nation’s honey-producing areas. Certified beekeepers provided honey samples, as well as information about floral origin, according to the locations where the beehives were situated and harvest season. In terms of floral type, honey samples came either from areas where hives were located within commercial eucalyptus plantations (*Eucalyptus* sp., *n* = 5), from native grasslands (*n* = 5) or from native forests (*n* = 5).

### 2.2. Physicochemical Analyses

Physicochemical analyses were performed according to the harmonised methods of the International Honey Commission [14]. The pH value of an aqueous honey solution (10 g of honey and 50 mL of CO_2_-free distilled water) was determined via potentiometry with a pH meter (Hanna HI 8424, HANNA Instruments, Sarmeola di Rubano PD, Italy). Moisture and total sugar content was determined via refractometry (ATAGO Hand Refractometer, Tokyo, Japan). For this analysis, only crystal-free and fluid honey samples that previously were homogenised and kept at 20 °C were used. Moisture content was expressed as grams of H_2_O per 100 g of honey, while that of sugar was expressed in °Brix.

Free, lactonic and total acidity were determined based on the potentiometric titration of an aqueous honey solution (10 g of honey and 75 mL of CO_2_-free distilled water) brought to a pH of 8.5 with 0.05 M NaOH. Next, 10 mL of 0.05 M NaOH was added to the alkaline solution, which then was back-titrated to pH 8.3 with 0.05 M HCl. Free, lactonic and total acidity values, expressed as milliequivalents per kilogram (meq/kg), were determined according to official methods of analysis [14].

### 2.3. C Isotopic Composition

Analyses of the C isotopic composition (δ^13^C) of the honeys and their proteins were performed according to the AOAC Official Method of Analysis [15]. Samples of filtered honey and protein were placed in tin capsules in an elemental analyser (Flash EA, 1112 series) coupled to an isotopic ratio mass spectrometer (Delta Plus Thermo Finnigan, Bremen, Germany) via a ConFlo III interface.

### 2.4. Statistical Analyses

Analysis of variance (ANOVA) and Tukey tests were carried out on all chemical variables. Multivariate DA was used to find the combination of independent variables (physicochemical and isotopic values) that minimised intragroup variance and maximised the variance between pre-established groups of honey. Using such a predictive model, it is possible to assign future samples to some of these groups according to their analytical values [16]. Both linear and quadratic DA procedures were evaluated. If the covariance matrices were the same for all groups, the model produced by linear DA was selected; otherwise, the quadratic DA was chosen [16].

The proportion of samples assigned to each of the original groups was one of the criteria used to evaluate the robustness of the DA model. This criterion also was applied to evaluate the cross-validation result; this stage consisted of a series of DA reruns (equal to the number of observations), with one of the samples omitted in each run. The closer this proportion to 1, the more robust the model. The process of cross-validation consisted in leaving a sample outside in each run; this involved separating the data in such a way that for each iteration there would be only one sample for the test data, with the rest forming the training data.

All statistical analyses were performed using the XLStat program (Addinsoft SARL, 2018).

## 3. Results and Discussion

### 3.1. Chemical Characterisation of Honeys

The quality of each honey was verified based on the various physicochemical parameters evaluated (pH; moisture; free, lactonic and total acidity; and sugar content), according to the Codex Alimentarius Commission [17] or MERCOSUR [18].

ANOVA revealed that a significant part of the variation in pH observed in the samples was related to their origin (*p* < 0.05). The increasing trend in pH among honey produced in native forest, eucalyptus forest and grassland (Table 1) is like that reported by Corbella and Cozzolino [5,10].

Comparisons outside of Uruguay of these types of environments can be taken only as a reference of product quality. Further, due to the unique botanical composition of Uruguayan grasslands and native forests compared with those of other countries, any international comparison can be carried out only for eucalyptus honey. The pH values obtained in this study for the honey samples derived from eucalyptus forest are similar to those of Argentinean honeys of the same floral origin [19,20]. This parameter thus can be considered a potential regional marker, considering the different values obtained for eucalyptus honey from India [21], Spain [22] and Morocco [23].

No statistical differences in moisture content were found between the honeys produced in the three Uruguayan environments (Table 1), these values were concordant with those obtained by Corbella and Cozzolino [5,10]. With respect to other regions, the mean moisture content of honey derived from the eucalyptus forest in this study is very close to that reported for Morocco [13,23] and Spain [22].

Neither free acidity nor lactic acidity nor total sugars (°Brix) differentiate between the origins of honey from Uruguay studied in this work (Table 1). In the case of eucalyptus honeys, presented values of free acidity coincide with Spanish [22] and Portuguese honeys [24], while the lactonic acidity and °Brix were slightly lower in the honeys of Uruguay than in Spanish [22] and Portuguese honeys [24].

In relation to ^13^C isotopic composition (Table 1), values did not vary statistically between honey groups. It should be noted, however, that the values are similar to those reported by Berriel and Perdomo for Uruguay [25]. Differences in honey and extracted protein δ^13^C values indicate that all the analysed honey samples were genuine according to White and Winters’ [15] criteria. In addition, the δ^13^C values recorded for the eucalyptus honeys and their proteins are like those of honey from Turkey [26] and South America [27].

### 3.2. Discrimination of Honey Origin

DA was used to identify the equations that could best differentiate honeys from the three production areas (eucalyptus forest, grasslands and native forest). The best model included the variables δ^13^C, pH, moisture and sugar content. The Box’s M test applied to the population equality of covariance matrices was not significant (*p* > 0.05), indicating that the data should be subjected to quadratic DA. However, although both Wilks’ lambda (0.089; *p* = 0.002) and Pillai’s trace (1.252; *p* = 0.004) values were significant, indicating that the DA model was acceptable, the final result was not good. Grassland and native forest centroids were located very near to each other (at −1.378 and −1.645 on the *x*-axis, and −0.921 and 0.868 on the *y*-axis, respectively), while model cross-validation produced error rate values for honey from the eucalyptus forest, grasslands and native forests of 0%, 60% and 20%, respectively, indicating that honeys from grasslands were confused with those from native forests, and vice versa.

In the next step, a group comprising samples of honey derived from grasslands and native forests was formed and then evaluated against honey originating from eucalyptus forests. According to this second analysis, values obtained for the Box’s M test (*p* < 0.0001), Wilks’ lambda (=0.099; *p* < 0.0001) and Pillai’s trace (=0.901; *p* < 0.0001) were all significant, indicating that the DA model was acceptable. Variables responsible for the most differentiation in the best model found were δ^13^C honey, °Brix, and moisture (Table 2). Only one quadratic discriminant function was associated with 100% of total variance, the intercept and coefficient of which are shown in Table 3. Centroids were 2.81 and −2.81 for honey from eucalyptus forests and from a natural and polyfloral environment (grasslands and native forests), respectively. The error rate of this model was 0%, both initially and after cross-validation (Table 4).

It was impossible to identify a robust DA model with which to classify Uruguayan honey according to its environment, based on the selected set of chemical variables. These results are likely due in part to the polyfloral origin of the grassland- and native forest-produced honeys. For this reason, a combined polyfloral honey group was formed and analysed against honey samples derived from eucalyptus forests, enabling the identification of a DA model with perfect classification ability. This model of classification was simple because it required only three variables that were easy to obtain during routine analyses (δ^13^C honey, °Brix, and moisture).

Many consumers have adopted the philosophy of acquiring food whose production system is sustainable. In this regard, it has been reported that apiaries located in monocultures are not sustainable as opposed to natural environments where several plant species coexist. The sustainability of hives in these types of contrasting environments depends on at least two factors: floral diversity and absence of pesticides [28,29].

In relation to the first factor, it has been found that the advance of afforestation as well as other monocultures had caused nutritional deficiencies in bees due to the loss of the diversity of pollen and nectar [28]. This deficiency results in bees that are more vulnerable to the attack of pathogens, and therefore, the number of individuals descends notoriously in these productive systems [28].

On the other hand, the use of herbicides and insecticides in agricultural areas based on monocultures [29] also affects the sustainability of beekeeping. The presence of pesticides may be minimal and not detected in honey; however, it has been seen that bees are subject to a cumulative effect of the minimum doses that have caused a significant mortality of bees [29].

Therefore, natural environments of honey production in Uruguay could be labelled as sustainable production and authenticated with the discriminant model proposed in this work. Authentication of honey, in this case, should involve the verification that the product matches the label statements [30].

## 4. Conclusions

These results suggest that it is possible to differentiate in Uruguay between honey-production systems: commercial plantations of eucalyptus vs natural environments such as grasslands and native forests. The proposed algorithm for the differentiation is based solely on the combination of composition variables of honeys’ δ^13^C, moisture and °Brix. That makes the differentiation quick and easy in the laboratory where further investigation can reveal if the honeys have been adulterated by adding high-fructose corn syrup.

In addition, it seems feasible to establish a certification of origin for honey from natural environments based on the DA analysis to respond to the concerns of some consumers interested in new product categories, such as sustainable production.

Finally, it is important to note that these results, although auspicious, are preliminary and need to be confirmed with other studies that include a more extensive database of Uruguayan honey.

## Figures and Tables

**Table 1:** Mean physicochemical properties of honey samples grouped by environmental origin.

Variable	Apiary Environment		
	Eucalyptus Forest (*n* = 5)	Native Forest (*n* = 5)	Grassland (*n* = 5)
	Mean ± SD ^1^		
pH	3.50 ± 0.34	3.86 ± 0.41	3.26 ± 0.39
Moisture (%)	17.84 ± 0.22	16.30 ± 0.54	15.96 ± 0.64
Free acidity (meq/kg)	27.76 ± 5.79	21.38 ± 7.47	29.81 ± 5.78
Lactonic acidity (meq/kg)	6.05 ± 3.03	3.42 ± 4.16	5.13 ± 2.72
Sugar content (°Brix)	79.7 ± 0.27	80.8 ± 0.45	81.10 ± 0.55
δ^13^C _Honey_ (‰)	−26.49 ± 0.53	−25.90 ± 0.40	−25.54 ± 0.45
δ^13^C _Protein_ (‰)	−25.75 ± 0.91	−25.72 ± 0.61	−25.57 ± 0.87

^1^ SD = standard deviation.

**Table 2:** Statistical data for variables used in the discriminant analysis.

Variable	Wilks’ Lambda	F	DF_1_^1^	DF_2_	*p*-Value
δ^13^C _Honey_	0.432	17.116	1	13	0.001
°Brix	0.266	35.951	1	13	<0.0001
Moisture	0.192	54.853	1	13	<0.0001

^1^ DF = degrees of freedom.

**Table 3:** Intercept and coefficients of the quadratic discrimination function.

Function’s Components	Honey Origin	
	E ^1^	P ^2^
Intercept	−1,311,343,810,475.940	−708,935.543
δ^13^C _Honey_	2.540	465.849
°Brix	25,712,623,826.863	15,142.429
Moisture	32,140,779,262.379	12,652.200
δ^13^C _Honey_ × δ^13^C _Honey_	−2.865	−2.763.
°Brix × °Brix	−126,042,275.087	−81.155
Moisture × Moisture	−196,941,054.823	−57.487
δ^13^C _Honey_ × °Brix	−2.712	−6.633
δ^13^C _Honey_ × Moisture	3.390	−4.404
Moisture × °Brix	−315,105,675.479	−134.786

Honey origin: ^1^ E = eucalyptus forest, ^2^ P = grassland + native forest.

**Table 4:** Classification of Uruguayan honey samples of different environmental origin and percentage of correctly classified observations using discriminant analysis.

Method	Predicted Group Membership (%)		
	Honey Origin		
	E ^1^	P ^2^	Overall
Original	100	100	100
Cross-validation	100	100	100

Honey origin: ^1^ E = eucalyptus forest, ^2^ P = grassland + native forest.
